# Recurrent Neuro-Renal Syndrome With Acute Kidney Injury From Anti-Pan-Neurofascin Antibody Resurgence: A Case Report

**DOI:** 10.1016/j.xkme.2026.101355

**Published:** 2026-04-06

**Authors:** David De Saint Gilles, Cédric Rafat, Jérôme Joël Devaux, Clémence Marois, Simon Leclerc, Tomoko Takano, Isabelle Brochériou, Nadine Elatram, François Husser, Laurent Mesnard, David Buob, Cyril Mousseaux

**Affiliations:** 1 Soins Intensifs Néphrologiques et Rein Aigu (SINRA), Assistance publique - Hôpitaux de Paris, Hôpital Tenon, Paris, France; 2 Sorbonne Université, Paris, France; 3 Institut de Génomique Fonctionnelle, Université de Montpellier, CNRS, INSERM, Montpellier, France; 4 Médecine Intensive et Réanimation à orientation Neurologique, Département de Neurologie, Assistance publique - Hôpitaux de Paris, Hôpital Pitié Salpêtrière Charles Foix, Paris, France; 5 Department of Medicine, Division of Clinical and Translational Research, Faculty of Medicine and Health Sciences, McGill University, Montréal, Québec, Canada; 6 Department of Medicine, Division of Nephrology, Research Institute of the McGill University Health Centre, Montréal, Québec, Canada; 7 Service d'Anatomie et Cytologie Pathologiques, Assistance publique - Hôpitaux de Paris, Hôpital Tenon, Paris, France

**Keywords:** Acute kidney injury, neurofascin, neuro-renal syndrome

## Abstract

We report the case of a patient with a severe neuro-renal syndrome characterized by acute-onset autoimmune nodopathy and nephrotic syndrome with acute kidney injury. The presence of IgG3 antibodies anti-pan-neurofascin (Nfasc155/186) confirmed the autoimmune nature of this pathology and justified treatment with plasma exchange, corticosteroids, tacrolimus, and subsequently rituximab. Kidney biopsy revealed focal segmental glomerulosclerosis lesions. This therapeutic approach led to complete remission of the clinical presentation. *APOL1* genotyping revealed G1/G1 alleles. One year after discontinuation of maintenance therapy with corticosteroids and tacrolimus, the patient experienced a relapse of the neuro-renal syndrome. This relapse was concomitant with the reappearance of anti-pan-neurofascin antibodies, which were undetectable during the first remission. Resumption of plasma exchange therapy guided by decreasing antibody titers along with corticosteroids and tacrolimus enabled neurologic remission, although chronic kidney disease persisted. Maintenance therapy with rituximab was initiated to prevent further relapses. This first reported case of late recurrence of anti-pan-neurofascin-associated neuro-renal syndrome highlights the importance of comprehensive immunologic and genetic evaluation, close immunomonitoring, and targeted immunotherapies, including plasma exchange and long-term rituximab maintenance.

## Introduction

In recent years, the pathophysiology of immune-mediated neurologic and nephrologic diseases, including conditions such as polyradiculoneuropathies and podocytopathies, has been redefined. Podocytopathies^1^ are characterized by podocyte injury, which manifests as nephrotic syndrome and sometimes acute kidney injury. Two main histologic subtypes are commonly recognized: minimal change disease and focal segmental glomerulosclerosis (FSGS). A key recent advance in podocytopathy research is the discovery of circulating autoantibodies targeting podocyte antigens (eg, anti-nephrin, anti-podocin, and anti-Kirrel1) in a significant subset of cases.^2^, ^3^, ^4^

Chronic inflammatory demyelinating polyradiculoneuropathy (CIDP) is a rare immune-mediated disorder of the peripheral nerves, typically causing combined motor and sensory deficits. Autoimmune nodopathies (ANs) constitute a related but distinct group defined by autoantibodies against nodal and paranodal adhesion molecules such as contactin-1, neurofascin-155 (and its isoforms Nfasc140/186), and Caspr1.^5^ Clinically, AN presents more acutely and severely than CIDP, often mimicking Guillain-Barré syndrome, and is marked by poor responsiveness to intravenous immunoglobulins.^6^

Over the last years, a steady number of CIDP cases have been described in association with nephrotic syndromes.^7^, ^8^, ^9^ This ‘neuro-renal’ autoimmune syndrome is partly explained by structural and molecular similarities shared by neurons and podocytes. At the histologic level, lesions consistent with podocytopathy and membranous nephropathy are described. Specifically, these cases are believed to be due to specific autoantibodies that may target common antigens expressed in both nerve tissue and podocytes (such as contactin-1^10^ and neurofascin^11^^,^^12^). This connection between neurons and podocytes is further reinforced by the description of certain genetic diseases associated with a neuro-renal phenotype.^13^

Although neuro-renal diseases are recognized as potentially responsive to immunosuppressive therapies, their natural course remains largely undefined. We report a case of relapse of AN, associated with nephrotic syndrome complicated by severe acute kidney injury, secondary to a resurgence of anti-pan-neurofascin antibodies and accompanied by FSGS lesions.

## The Case

We describe the case of a 74-year-old male patient with the only significant medical history being hypertension, with no known chronic kidney disease.

### First Episode

The patient was admitted to the intensive care unit with acute polyneuropathy, presenting predominantly with motor deficits in all 4 limbs, accompanied by areflexia and paresthesia. A lumbar puncture revealed no cells or pathologic levels of protein in cerebrospinal fluid (protein level 0.48 g/L). Electromyography was consistent with acute motor sensory axonal neuropathy. The patient’s motor function rapidly deteriorated, with the onset of diaphragmatic and bulbar involvement leading to orotracheal intubation.

**Figure 1:**
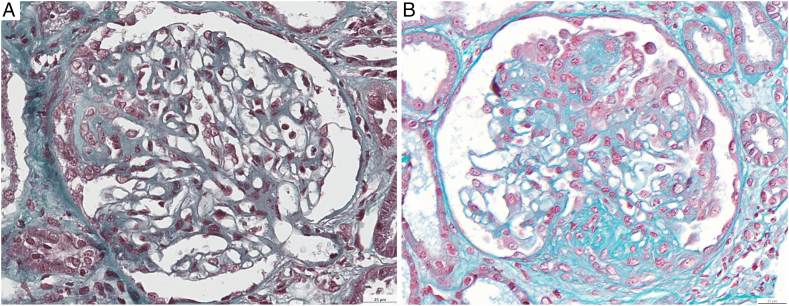
Renal histological findings. The first biopsy (A) shows a predominance of FSGS with a ‘tip lesion’ subtype (Masson's trichrome stain). The second biopsy (B) shows a predominance of FSGS with a ‘NOS lesion’ subtype (Masson's trichrome stain). FSGS, focal segmental glomerulosclerosis; NOS, not otherwise specified. Scale bar = 25 μm.

The patient simultaneously developed an acute kidney injury (creatinine peak 3.5 mg/dL), nephrotic syndrome with hypertension. The urinary protein-creatinine ratio (UPCR) was 3.3 g/mmol, and albuminemia was 16 g/L. Immunologic testing, including ANCA, anti-GBM, anti-PLA2R/THSD7A, and ANA, was negative. A first kidney biopsy (Fig 1A) revealed FSGS with a ‘tip lesion’ subtype. Mild interstitial fibrosis (15%) and acute tubular necrosis were present. No immune deposits were found on immunofluorescence. Whole exome sequencing revealed homozygosity for the *APOL1* G1/G1 risk alleles.

Serum testing revealed the presence of anti-pan-neurofascin antibodies, predominantly of the IgG3 isotype, targeting the Nfasc155 and Nfasc186 isoforms, with titers of 1:6,000. Anti-contactin-1 and CASPR1 antibody tests were negative.

**Figure 2:**
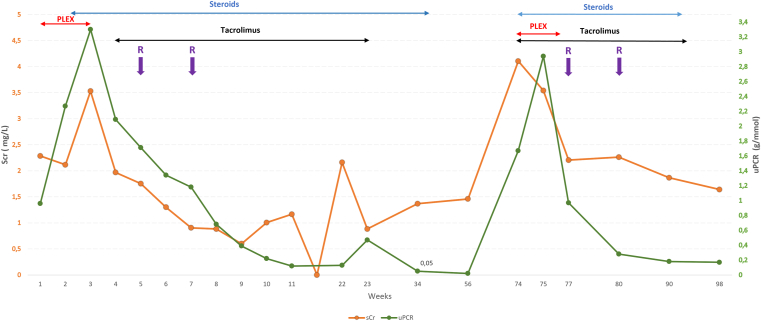
Evolution of creatinine and uPCR (1 value per week) with treatments. PLEX, plasma exchange; R, rituximab; Scr, serum creatinine; uPCR, urinary protein-creatinine ratio.

The patient underwent plasma exchange (PLEX) and received corticosteroid therapy (1 mg/kg). Given the prolonged recovery, tacrolimus (target 8-10 ng/mL) and 2 doses of rituximab 15 days apart were added (Fig 2). Weaning from ventilation was completed on day 56. Kidney function improved, and anti-pan-neurofascin antibodies were no longer detectable.

On day 71, he was discharged to rehabilitation with residual proximal limb weakness. Corticosteroids were tapered to 10 mg by 6 weeks. Renal function stabilized with an estimated glomerular filtration rate 90 mL/min/1.73 m^2^ and UPCR 0.12 g/mmol.

After good neurologic recovery (walking with a walker) and partial renal remission (estimated glomerular filtration rate 64 mL/min/1.73 m^2^, UPCR 0.15 g/mmol), immunosuppressive therapy was stopped.

### Second Episode

One year after stopping immunosuppressive therapy, the patient experienced sudden muscle weakness in all limbs, paresthesia, sensory disturbances, and generalized edema. Investigations revealed a relapse of the neuro-renal syndrome with acute kidney injury (peak creatinine 4.1 mg/dL), recurrent nephrotic syndrome (peak UPCR 4.4 g/mmol, including 80% albumin), and a new episode of acute motor sensory axonal neuropathy (confirmed by electromyography).

Anti-pan-neurofascin antibodies were again strongly positive: anti-Nfasc155 antibody titer was 1:1,400 and the anti-Nfasc186 titer was 1:2,600 (with IgG3 isotype predominant). A second kidney biopsy (Fig 1B) revealed FSGS lesions of the ‘not otherwise specified’ subtype. Interstitial fibrosis was now estimated at 50%. Electron microscopy showed diffuse foot process effacement. Serum testing for anti-nephrin autoantibodies was negative.

Management included PLEX, high-dose corticosteroids, and tacrolimus. Marked neurologic improvements were observed on a daily basis, particularly after each PLEX session. PLEX was discontinued once anti-pan-neurofascin titers became negative (after 7 sessions) and a new cycle of rituximab was administered.

After 20 days, he was discharged with resolved sensorimotor deficits, stable renal function (estimated glomerular filtration rate 32 mL/min/1.73 m^2^), and persistent proteinuria (0.84 g/mmol). At 6 months, renal function was stable (estimated glomerular filtration rate 44 mL/min/1.73 m^2^), proteinuria was improved (0.17), and neurologic symptoms had fully resolved. Rituximab maintenance was continued alone (1 g every 5-6 months).

## Discussion

This is the first report of a late relapse of a neuro-renal syndrome combined with nephrotic syndrome with FSGS lesions and an AN with anti-pan-neurofascin antibodies.

Neurofascin belongs to the L1 family of transmembrane cell adhesion molecules. Three main isoforms are expressed in the nervous system: Nfasc155, Nfasc140, and Nfasc186. Nfasc155 is expressed by glial cells in the paranodal regions along the axon, whereas Nfasc140 and Nfasc186 are expressed by neurons at the nodes of Ranvier and the axon initial segments.^14^^,^^15^ Certain subtypes of AN are associated with the presence of anti-Nfasc155, anti-Nfasc186, and anti-Nfasc140 autoantibodies.^11^^,^^16^ Although historically considered neuron-specific, Nfasc186 has also been shown to be expressed by podocytes.^12^

Other cases of neuro-renal syndromes have been reported, involving various combinations of CIDP and glomerular diseases such as minimal change disease, FSGS, or membranous nephropathy.^8^^,^^10^^,^^11^^,^^17^ Although the involvement of anti-Nfasc155 antibodies is relatively well documented in AN, these antibodies are not typically associated with renal involvement and are mostly of the IgG4 subclass. Anti-Nfasc186–associated AN was first reported in 5 patients (4 IgG4, 1 IgG3), 2 of whom developed nephrotic syndrome (without further clinical or histologic details).^11^ The expression of Nfasc186 in podocytes may explain the simultaneous glomerular and neurologic involvement in these patients.

The severity of the clinical presentation may also be explained both by the development of IgG3 subclass antibodies, which are known to activate the classical complement pathway, and by the presence of anti-pan-neurofascin antibodies, which are associated with more severe neurologic manifestations.^18^ One key takeaway message is to systematically search for proteinuria during the initial phase of an AN.

Other mechanisms, such as epitope spreading (where the original antigenic scope of the autoimmune response expands to adjacent antigens over time) and molecular mimicry (where a neural epitope closely resembles a kidney epitope, which is then targeted), might be at play. Accordingly, anti-nephrin autoantibodies were excluded. However, newly identified anti–slit diaphragm autoantibodies may also be involved.^3^^,^^4^ To fully establish the implication of anti-pan-neurofascin IgG in renal involvement, the pathogenicity of anti-Nfasc186 autoantibodies has yet to be demonstrated on kidney tissue. Expression of Nfasc186 in podocytes appears to be sufficient to explain the nephrotic syndrome and neurologic phenotype observed in these patients. Furthermore, the strong correlation between relapse and the reappearance of the autoantibodies further reinforces their pivotal role in neuro-renal syndrome pathogenesis.

The severe renal involvement observed in our patient may also be attributable to the presence of a high-risk *APOL1* genotype, as he was found to carry the homozygous G1/G1 *APOL1* variant. A high-risk *APOL1* genotype has been shown to contribute to chronic kidney disease and hypertension, as well as to the development of collapsing FSGS, particularly when accompanied by additional ‘second hits,’ such as HIV, parvovirus B19, or SARS-CoV-2.^19^ Here, AN may have acted as a second hit, promoting the development of FSGS.

This late relapse underscores the risk of recurrence in anti-pan-neurofascin–mediated AN and highlights the need for close clinical and immunological monitoring. It may warrant consideration of long-term immunosuppression, possibly with rituximab, although follow-up and treatment strategies cannot be standardized based on a single case.

The discovery of anti-nephrin antibodies has recently transformed the field of podocytopathies, elucidating the pathogenesis of a subset of podocytopathies and opening new avenues for antibody-targeted therapies.^2^ In addition to corticosteroids, both PLEX (to address circulating autoantibodies) and anti-CD20 therapy (given the reduced steroid responsiveness observed in these cases) represent plausible therapeutic approaches. To expedite the clearance of these autoantibodies, urgent administration of imlifidase (an endopeptidase that cleaves the constant fragment of immunoglobulins) emerges as a promising adjunctive option. This strategy is compelling in neuro-renal syndromes, where respiratory and bulbar muscle involvement carry life-threatening risks. Accordingly, a randomized controlled trial of imlifidase in Guillain-Barré syndrome has recently been completed (NCT03943589). Another potential therapeutic target is the FcRn antagonist efgartigimod, which is currently being investigated for CIDP but has not yet been tested for nephropathies.^20^

This report documents the first known relapse of neuro-renal syndrome driven by anti-pan-neurofascin antibodies. The severity of the disease course underscores the need for comprehensive immunologic and genetic evaluation, vigilant monitoring, and the use of targeted immunotherapies such as PLEX and rituximab.
